# Vertigo During Pregnancy: A Narrative Review of the Etiology, Pathophysiology, and Treatment

**DOI:** 10.7759/cureus.55657

**Published:** 2024-03-06

**Authors:** Zlatko Kirovakov, Asen Kutsarov, Svetoslav Todorov, Plamen Penchev

**Affiliations:** 1 Department of Obstetrics and Gynaecology, University Hospital for Active Treatment – Burgas, Burgas, BGR; 2 Faculty of Public Health and Health Care, Prof. Asen Zlatarov University, Burgas, BGR; 3 Department of Health Care, Medical University Varna, Affiliate Veliko Tarnovo, Veliko Tarnovo, BGR; 4 Department of Neurological Surgery, University Hospital for Active Treatment – Burgas, Burgas, BGR; 5 Faculty of Medicine, Prof. Asen Zlatarov University, Burgas, BGR; 6 Faculty of Medicine, Medical University of Plovdiv, Plovdiv, BGR

**Keywords:** treatment, pathophysiology, etiology, prisma guidelines, pregnancy, vertigo

## Abstract

From the time of conception until the time of labor, a woman's body and mind undergo a variety of hormonal and other changes. Patients may also experience vertigo and a lack of balance during this period. Disabling and physically painful, these symptoms may strike at any moment. Pregnancy-related vertigo has been the focus of several studies. We looked at the research on vertigo in pregnant women in detail. This narrative review aims to examine the causes, pathophysiology, and current treatments for vertigo during pregnancy. Vertigo during pregnancy has a diverse etiology, with typical causes including hormonal changes and modifications in vascular dynamics. Vertigo may start to appear due to pathophysiological mechanisms involving vestibular and central nervous system adaptations. Numerous alternatives for treatment are available, including dietary changes, vestibular therapy, medicines, and surgical procedures. The thorough assessment of the current research on vertigo during pregnancy provided by this narrative review will help medical practitioners make wise clinical decisions.

## Introduction and background

The physiological state of pregnancy in a female is quite significant due to the impact of hormones, the cardiovascular system, and the changes in the mind throughout pregnancy [1]. The circulatory, respiratory, gastrointestinal, musculoskeletal, dermatological, and auditory vestibular systems all undergo structural and functional changes as a consequence of specific hormones such as progesterone, estrogen, placental lactogen relaxing, and human chorionic gonadotropin [2]. The audiovestibular system is implicated in several symptoms, including hearing loss, tinnitus, facial nerve paralysis, otosclerosis, autophony, and vertigo, which may appear at the starting time or get worse during pregnancy. Some pregnant people have vertigo throughout their pregnancies, which may be a frequent and upsetting condition [3,4]. Vertigo is characterized by a sensation of spinning or dizziness and is also characterized by a sense of instability or unsteadiness [5]. As beautiful and life-altering as pregnancy is, it may bring a variety of physical changes, including those that have an impact on balance and the vestibular system [6,7]. These shifts may exacerbate vertigo symptoms in some pregnant mothers. It's crucial to understand that vertigo during pregnancy is common and may have several reasons. There may be physiological changes at play, like shifts in hormone levels, shifts in blood pressure [8,9], and an increase in blood volume in the body. Some conditions like “Benign Paroxysmal Positional Vertigo (BPPV)”, an inner ear disorder, may become worse when pregnant [10,11]. The etiology of vertigo in pregnant women also needs careful examination [12]. The severity and onset of vertigo symptoms may be affected by modifications in inner ear function, hormone changes, and other physiological changes [13]. To design effective, tailored treatment regimens, knowledge of these routes is crucial.

This narrative review's management of vertigo during pregnancy is a key component [14,15]. Pregnant women with vertigo require safe, scientifically supported treatments to reduce their symptoms without endangering their own or their unborn child's health. Evaluation of the available literature on treatment methods, such as drugs, exercises for vestibular rehabilitation, and lifestyle changes, will provide important insights into the most effective ways to handle vertigo during pregnancy. We want to add the body of knowledge that promotes healthy pregnancies and improves the quality of life for expecting moms dealing with this difficult condition by looking at the etiology, pathophysiology, and therapeutic choices.

The article investigated the etiological factors that contribute to BPPV and the function of pregnancy-related changes in BPPV [16]. It featured four expectant ladies who had their initial BPPV diagnosis during pregnancy. According to the research every part of a pregnant woman's body, including her senses is affected by the myriad physiological changes she experiences [17]. The goals of the review provided an overview of the available evidence and treatment options for women with audiovestibular problems during pregnancy [18]. The goal of the study was to increase awareness of the problem and provide the doctor with a summary of the most recent findings about diagnosis, clinical treatment, and pathophysiology [19]. The article aimed to conduct a literature review on the pharmacological profile of betahistine, and the article was to conduct a literature review on the pharmacological profile of betahistine as well as the data supporting its efficacy and safety in the management of peripheral vertigo [20]. In addition to this, an up-to-date discussion of pharmacokinetics, mechanisms of action, and pharmacodynamics was included in the review.

## Review

Etiology and pathophysiology

The development of vestibular symptoms like vertigo has been linked to the physiological changes that occur during pregnancy as a potential substrate [21]. Particular ear abnormalities in pregnancy have been linked to particular pathophysiological pathways, illnesses like “Meniere's, sudden sensorineural hearing loss, blocked eustachian tubes, benign paroxysmal positional vertigo". There are also direct effects of pregnancy hormones on the labyrinthine system, such as fluid retention in the endolymph and perilymph, immune system modulation that causes virus reactivation, and hypercoagulability; one of the most common discoveries in medicine is endolymphatic hydrops. Early pregnancy increases the severity of vertigo in Meniere's disease (MD) because of the lower serum osmolality. Although the exact cause of the lower serum osmolality in early pregnancy is uncertain, it worsens vertigo attacks in MD. The severity of endolymphatic hydrops is increased when there is a sudden reduction in serum osmolality that creates a differential in osmotic pressure between the sac's outside and inner layers, allowing free water to enter the sac from the outside. Pregnancy seems to be a suitable experimental setting for examining how serum osmolality relates to MD. It's unclear the BPPV causes pathophysiology during pregnancy. About half of people with BPPV had a head injury as a contributing cause of their condition [22].

Otoliths in any of the three semicircular canals, either floating freely (canalolithiasis) or stuck to the cupula (cupulolithiasis) are the most prevalent cause of BPPV [23]. People with BPPV have basophilic deposits in the cupula of the lowest crista of the posterior semicircular canal in their temporal bones [23]. These deposits were assumed to reflect degenerated otoconia originating from the utricular macula. The cupula deposits were thought to transform the sensory organ into a gravity receptor that was triggered during the Hallpike maneuver. Vertigo and nystagmus were relieved after choosing to denervate the posterior semicircular canal sensory organ of the lowest labyrinth [24]. Free-floating particles have been seen in the limb of the canal in patients having closure of the lateral hemispherical canal for BPPV [24]. The two types of deposits that may form in the posterior semicircular canal are called cupulolithiasis and canalolithiasis, respectively.

The etiology of BPPV is assumed to be prolonged bed rest. Recently, there have been studies claiming that problems with the metabolism of calcium and vitamin D increase the incidence of BPPV [24,25]. Pregnancy affects the metabolism of calcium and vitamin D, especially in the latter trimesters due to the fetus's fast development [25]. This might be a significant risk factor for pregnant women who have BPPV. There are several etiologies for BPPV; however, the etiology in pregnancy cannot be explained by hormonal imbalances or alterations. During menstruation, pregnancy, and menopause, hormone fluctuations have various impacts on homeostasis and metabolism. There have been several suggestions regarding the effects of estrogen on ear functioning in normal mice containing estrogen receptors in their inner ears. Spiral ganglion and stria vascularis contain these receptors, which are essential for sound transmission and maintaining the homeostasis of the inner ear. Changes in estrogen levels are hypothesized to reduce the electrolyte content of endolymphatic fluid, which leads to the decline in otoconial fibers or elevated endolymphatic pH due to a variety of causes, which leads to the degeneration of otoconial fibers. By regulating ion and anion channels, estrogen is hypothesized to have an impact on the endolymphatic ionic and anionic balance [26].

However, varying glucose and lipid metabolism estrogen also improves the vascular supply to the macula and otoconia. Pregnancy affects the metabolism of calcium and vitamin D, especially in the final trimester because of the fetus's fast development. Having been diagnosed with BPPV during pregnancy, this is a significant risk factor [27,28]. By maintaining the calcium concentration in the vestibular endolymph at a normal critical level, since high or low calcium would result in aberrant otoconia, the normal blood vitamin D content is necessary for the formation of normal otoconia. This is accomplished through the labyrinth's epithelial calcium channel transport system, which is supported by vitamin D receptors. Vitamin D insufficiency leads to the formation of aberrant otoconia, which culminates in otolith dysfunction [29].

Clinical manifestations

A woman's pregnancy is a crucial physiological stage of her life and any symptoms should be considered to prevent any potential harm to the mother or baby. Vomiting and nausea during pregnancy are often physiological [30]. A patulous eustachian tube, nasal congestion, nasal hemorrhage, gingivitis, and reflux esophagitis are a few otolaryngological signs that may be made worse by certain endocrinological, metabolic, and physiologic abnormalities [31,32]. Vertigo attacks are triggered by head movement in BPPV [33]. When the patient turns over onto the afflicted side or tilts their heads back while facing upward, they have vertigo episodes. Associated symptoms, including nausea and vomiting, may also exist. Frequently, patients complain of nausea, vomiting, and dizziness. In the case of MD, hearing loss and tinnitus may also be present. In MD, vertigo may last anywhere from 20 minutes and 20 hours. The mucosal membrane of the nose and nasopharynx changes as a result of estrogen and progesterone usage. It leads to eustachian tube malfunction and displays symptoms of ear blockage and autophony during pregnancy. It also causes nasal discharge and nasal obstruction. These symptoms are momentary and completely disappear following delivery. Women who have MD during pregnancy often have episodic vertigo, variable sensorineural hearing loss, and roaring or hissing tinnitus [34].

Treatment

Treatment for vertigo during pregnancy is difficult. It is possible to avoid taking appropriate drugs to avoid harming the fetus [35]. The majority of BPPV sufferers self-limit their symptoms. For BPPV, the canal repositioning operation is regarded as an effective and time-tested noninvasive therapy approach. Typically, the Epley technique and Semont's maneuver are used to treat BPPV [36]. Regarding their effectiveness outside of central habituation, there is significant debate currently going on. A serious issue might arise from certain resistant BPPV instances and illness variations. Pregnancy-specific medical care is suitably cautious. Reducing salt and caffeine intake is advised as a therapy for MD during pregnancy [37]. In medical therapy, lowering endolymphatic pressure is always the goal [38]. The antipsychotic effects of prochlorperazine, such as additional pyramidal effects in the infant if taken in the third trimester, must be used during pregnancy with care since it might trigger an abrupt bout of vertigo. Dimenhydrinate (Dramamine) and meclizine are safe when used in low dosages to treat vertigo in MD patients. The MD may be well-controlled with isosorbide as well. Typically, isosorbide is a safe medicine to use while pregnant [39]. In medicine, intratympanic therapy with steroids via the round window is intended to have an acceptable impact while avoiding systemic drugs' negative side effects.

The saccular, inferior vestibular nerve and central connections may be tested to see whether they are functioning correctly using a procedure called vestibular-evoked myogenic potential (VEMP) [40]. An effective vestibular suppressor is diazepam. However, its use during pregnancy is not advised due to possible adverse consequences, including floppy baby syndrome and benzodiazepine withdrawal syndrome [41]. As an anti-inflammatory, corticosteroids are useful in treating vestibular disorders such as vestibular neuritis, reducing nerve inflammation, and hastening the recovery of vestibular deficit. To encourage fetal lung development, dexamethasone and/or betamethasone may be administered to expectant mothers at risk for preterm delivery [42].

For individuals with vestibular neuritis, Cawthorne-Cooksey exercises are particularly effective for at-home exercise. Standing on a platform that is moving in anteroposterior or mediolateral directions about the participants is one of the workouts. For five days, these exercises should be performed twice daily for 30 minutes at a time [43]. The Epley maneuver is the canalith repositioning technique that is most employed. To improve circulation; patients should avoid standing for extended periods and make sure to move about when they are. In order to prevent BPPV, vitamin D3 supplements must be given to pregnant women [44,45].

## Conclusions

Pregnant women may have severe pain and incapacity as a result of these changes, which may cause rapid, unexpected bouts of vertigo and instability. Fortunately, the analysis found several effective treatments for vertigo during pregnancy. Among these alternatives are dietary changes, vestibular treatment, medication therapies, and even surgical operations. The choice of therapy should be made by the unique circumstances of the patient, taking into consideration the severity of the symptoms, underlying reasons, and possible hazards to both the mother and the growing baby.
